# Disparities in Excess Mortality Associated with COVID-19 — United States, 2020

**DOI:** 10.15585/mmwr.mm7033a2

**Published:** 2021-08-20

**Authors:** Lauren M. Rossen, Farida B. Ahmad, Robert N. Anderson, Amy M. Branum, Chengan Du, Harlan M. Krumholz, Shu-Xia Li, Zhenqiu Lin, Andrew Marshall, Paul D. Sutton, Jeremy S. Faust

**Affiliations:** ^1^National Center for Health Statistics, Hyattsville, Maryland; ^2^Yale University School of Medicine, Center for Outcomes Research and Evaluation, New Haven, Connecticut; ^3^Department of Emergency Medicine, Brigham and Women’s Hospital, Harvard Medical School, Boston, Massachusetts.

The COVID-19 pandemic has disproportionately affected Hispanic or Latino, non-Hispanic Black (Black), non-Hispanic American Indian or Alaska Native (AI/AN), and non-Hispanic Native Hawaiian or Other Pacific Islander (NH/PI) populations in the United States. These populations have experienced higher rates of infection and mortality compared with the non-Hispanic White (White) population (*1*–*5*) and greater excess mortality (i.e., the percentage increase in the number of persons who have died relative to the expected number of deaths for a given place and time) (*6*). A limitation of existing research on excess mortality among racial/ethnic minority groups has been the lack of adjustment for age and population change over time. This study assessed excess mortality incidence rates (IRs) (e.g., the number of excess deaths per 100,000 person-years) in the United States during December 29, 2019–January 2, 2021, by race/ethnicity and age group using data from the National Vital Statistics System. Among all assessed racial/ethnic groups (non-Hispanic Asian [Asian], AI/AN, Black, Hispanic, NH/PI, and White populations), excess mortality IRs were higher among persons aged ≥65 years (426.4 to 1033.5 excess deaths per 100,000 person-years) than among those aged 25–64 years (30.2 to 221.1) and those aged <25 years (−2.9 to 14.1). Among persons aged <65 years, Black and AI/AN populations had the highest excess mortality IRs. Among adults aged ≥65 years, Black and Hispanic persons experienced the highest excess mortality IRs of >1,000 excess deaths per 100,000 person-years. These findings could help guide more tailored public health messaging and mitigation efforts to reduce disparities in mortality associated with the COVID-19 pandemic in the United States,* by identifying the racial/ethnic groups and age groups with the highest excess mortality rates.

Data on the weekly number of deaths from all causes and from COVID-19 that occurred during December 29, 2019–January 2, 2021, were obtained from the National Vital Statistics System (NVSS).^†^ These data included all deaths occurring in the 50 U.S. states and District of Columbia (DC) and were not limited to U.S. residents; approximately 0.2% of decedents overall were foreign residents. Deaths attributed to COVID-19 were identified with the *International Classification of Diseases, Tenth Revision,* *Clinical Modification* (ICD-10-CM) code U07.1, which indicated that COVID-19 was an underlying or contributing cause of death.^§^ Observed numbers of deaths were weighted to account for incomplete reporting by jurisdictions^¶^ (50 states and DC) in the most recent weeks, where the weights were estimated based on the completeness of provisional data from 2019 (*7*).

Annual population estimates from the U.S. Census Bureau** by age group and race/ethnicity for 2015–2019 were projected using seasonal autoregressive integrated moving average (sARIMA) models to obtain weekly estimates through 2020. The weekly expected numbers of deaths were estimated using sARIMA models of the all-cause mortality IRs (deaths per 100,000 person-weeks) based on 2015–2019 data multiplied by the weekly adjusted population counts for the period December 29, 2019–January 2, 2021. The resulting weekly expected numbers of deaths were subtracted from the observed numbers of deaths to generate estimates of excess deaths. The weekly population denominators were adjusted for the cumulative numbers of excess deaths in each age and racial/ethnic group occurring through each week of 2020,^††^ to account for smaller than expected population growth through the COVID-19 pandemic because of the number of excess deaths that occurred during that time.

Weekly percentage excess mortality (excess deaths as a percentage of expected deaths) and quarterly or annual excess mortality IRs (number of excess deaths per 100,000 person-quarters or person-years for 2020 overall) were estimated by age group (<25 years, 25–64 years, and ≥65 years) and race/ethnicity group (i.e., Asian, AI/AN, Black, Hispanic, NH/PI, and White populations). Persons of more than one race or for whom race and ethnicity were unknown are not shown separately because numbers were small. R statistical software (version 4.0.2; R Foundation) was used to conduct the analyses. These activities were reviewed by CDC and conducted consistent with applicable federal law and CDC policy.^§§^

Among persons aged <25 years, the percentage excess mortality was highly variable throughout 2020 for most racial/ethnic groups. Among persons aged 25–64 years, the largest percentage excess mortality occurred among Hispanic persons, with three distinct peaks of ≥75% in April, July, and December 2020. In this age group, among AI/AN persons, percentage excess mortality peaked at ≥60% during June–July and December 2020; and peaked among Black persons in April 2020 at approximately 70%, with a second smaller peak (40%) in July 2020. Among Asian persons, peaks of at least 75% occurred in April and December 2020; and among NH/PI persons, percentage excess mortality peaked in July and December 2020, at nearly 90%. Percentage excess mortality among White persons aged 25–64 years was relatively consistent (approximately 15% to 20%) during April–December 2020 (Figure 1).

**FIGURE 1:**
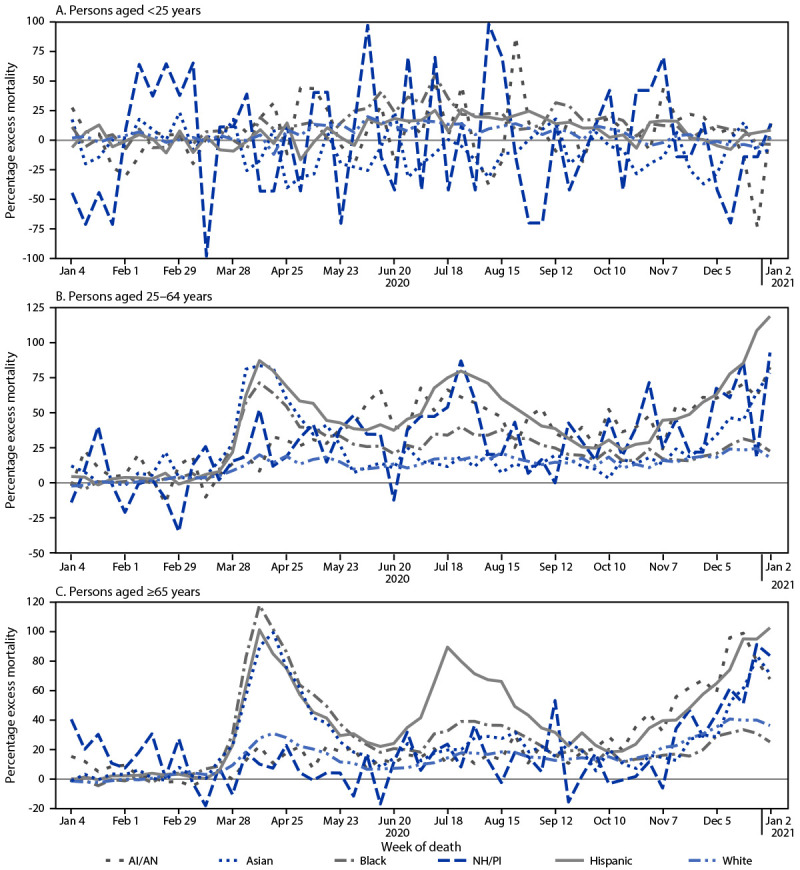
Weekly percentage excess all-cause mortality* for persons aged <25 years (A), 25–64 years (B), and ≥65 years (C), by race/ethnicity^†^ — United States, 2020 **Abbreviations:** AI/AN = American Indian/Alaska Native; NH/PI = Native Hawaiian/Other Pacific Islander; sARIMA = seasonal autoregressive integrated moving average. * Weekly numbers of deaths from all causes by age group and race/ethnicity were obtained from the National Vital Statistics System. The expected numbers of deaths were estimated using sARIMA models of weekly all-cause mortality incidence rates (deaths per 100,000 population-weeks) from 2015–2019, multiplied by the weekly population projections during December 29, 2019–January 2, 2021. The percentage excess corresponds to the number of excess deaths divided by the expected number of deaths. Weeks 1–53 of 2020 are shown. The scale of the y-axis differs for each age group. ^†^ AI/AN, Asian, Black, NH/PI, and White persons were non-Hispanic; Hispanic persons could be of any race.

Among persons aged ≥65 years, percentage excess mortality among Hispanic persons peaked at ≥90% in April, July, and December 2020. Among AI/AN and NH/PI persons in this age group, percentage excess mortality peaked at nearly 100% in December 2020, and among Asian persons, peaks of 100% and >80% were seen in April and December 2020, respectively. For Black persons aged ≥65 years, percentage excess mortality peaked in April 2020, at nearly 120%, with smaller peaks of 38% in July 2020 and 32% in December 2020. Percentage excess mortality among White persons aged ≥65 years peaked at 29% in April 2020 and 39% in December 2020 (Figure 1).

Black persons had the highest excess mortality IR among all persons aged <25 years with 14.1 excess deaths per 100,000 person-years in 2020, followed by AI/AN persons (6.5). Among adults aged 25–64 years, the highest total excess mortality IR was among AI/AN persons (221.1), followed by Black (133.4), NH/PI (124.9), Hispanic (98.5), White (51.2) and Asian persons (30.2). Quarterly excess mortality IRs among persons aged 25–64 years fell from April–June 2020 to October–December 2020 among Black persons, were relatively flat for Hispanic and Asian persons, and rose for AI/AN, NH/PI, and White persons. Among adults aged ≥65 years, the largest excess mortality IRs were seen among Black and Hispanic persons (1,033.5 and 1,007.0, respectively), followed by AI/AN (650.0), White (500.1), Asian (483.7), and NH/PI persons (426.4). Among Asian and Black adults aged ≥65 years, excess mortality IRs peaked during April–June and declined thereafter. Among Hispanic adults in this age group, excess mortality IRs were stable from April–June 2020 through October–December 2020; quarterly excess mortality IRs were highest in October–December 2020 for AI/AN, NH/PI, and White adults aged ≥65 years (Figure 2).

**FIGURE 2:**
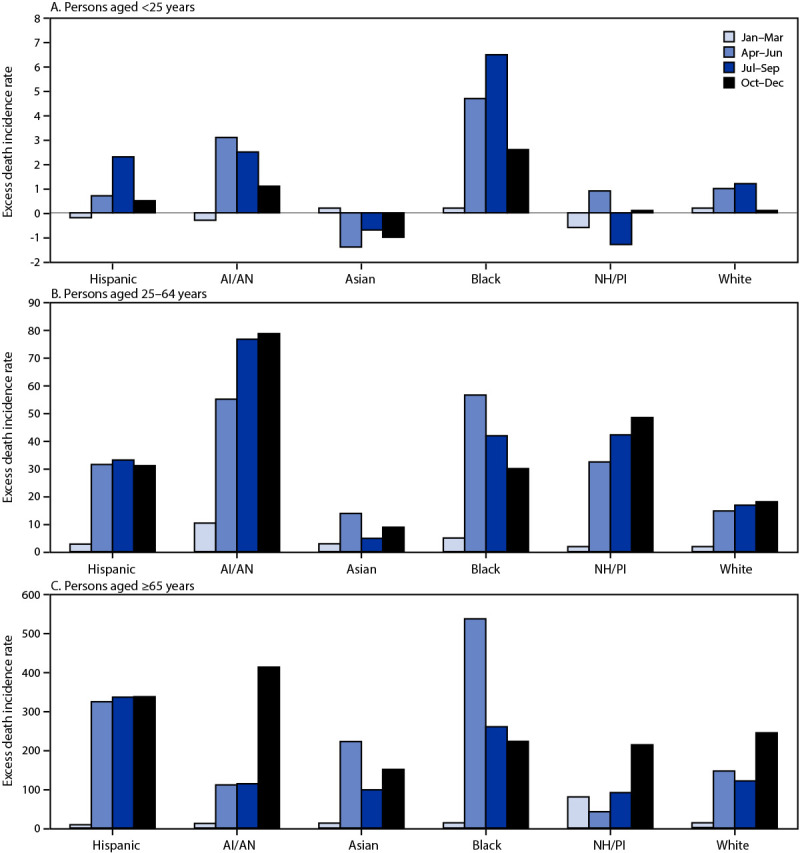
Quarterly excess all-cause mortality incidence rates* and annual excess incidence rates^†^ for persons aged <25 years (A), 25–64 years (B), and ≥65 years (C), by race/ethnicity^§^ — United States, 2020^¶^** **Abbreviations:** AI/AN = American Indian/Alaska Native; IR = incidence rates; NH/PI = Native Hawaiian/Other Pacific Islander. * Excess deaths per 100,000 person-quarters. ^†^ Annual excess death IRs for Hispanic, AI/AN, Asian, Black, NH/PI, and White persons were as follows: aged <25 years: 3.3, 6.5, −2.9, 14.1, −1.0, and 2.2, respectively; aged 25–64 years: 98.5, 221.1, 30.2, 133.4, 124.9, and 51.2, respectively; aged ≥65 years: 1,007.0, 650.0, 483.7, 1,033.5, 426.4, and 500.1, respectively. ^§^ AI/AN, Asian, Black, NH/PI, and White persons were non-Hispanic; Hispanic persons could be of any race. ^¶^ Weeks 1–52 (week 53 omitted to ensure each quarter consisted of 13 weeks and the four quarters summed to the total). The scale of the y-axis differs for each age group. ** Negative excess mortality IRs mean that there were fewer deaths than expected for that group.

Among persons aged <25 years, the percentage of excess mortality directly attributed to COVID-19 during 2020 ranged from 9.8% (Black persons) to 34.3% (AI/AN persons) (Table). Among those aged 25–64 years, the percentage ranged from 46.4% (White persons) to 79.2% (NH/PI persons). Among persons aged ≥65 years, the percentage ranged from 78.7% (Black persons) to 123.8% (AI/AN persons).

**TABLE:** Total number of excess deaths^*^ and percentage of total excess deaths that were directly attributed to COVID-19, by age group and race/ethnicity^†^ — United States, 2020

Age group, yrs	Race/Ethnicity	No. of excess deaths (% directly attributed to COVID-19)
**<25**	Hispanic	857 (33.7)
	American Indian or Alaska Native	58 (34.3)
	Asian	−158 (NA)
	Black	1,983 (9.8)
	Native Hawaiian or Other Pacific Islander	−2 (NA)
	White	1,299 (13.6)
**25–64**	Hispanic	32,305 (77.7)
	American Indian or Alaska Native	2,950 (61.2)
	Asian	3,613 (76.8)
	Black	30,035 (57.1)
	Native Hawaiian or Other Pacific Islander	447 (79.2)
	White	54,197 (46.4)
**≥65**	Hispanic	52,132 (85.0)
	American Indian or Alaska Native	2,215 (123.8)
	Asian	13,554 (80.6)
	Black	55,004 (78.7)
	Native Hawaiian or Other Pacific Islander	304 (109.4)
	White	223,995 (93.2)

**Abbreviations: **IRs = incidence rates; NA = not applicable.

* Weekly numbers of deaths from all causes by age group and race/ethnicity were obtained from the National Vital Statistics System. The expected numbers of deaths were estimated using seasonal autoregressive integrated moving average models of weekly all-cause mortality IRs (deaths per 100,000 population-weeks) during 2015 through 2019, multiplied by the weekly population projections during December 29, 2019–January 2, 2021. The total number of excess deaths corresponded to the sum of the observed number of deaths minus the expected number of deaths during 2020. Negative numbers of excess deaths mean that fewer deaths occurred than were expected for that group; for these values, not applicable is shown for the percentage of excess deaths attributed to COVID-19. Values of >100% mean that the number of COVID-19 deaths exceeds the number of excess deaths in that group. Data include weeks 1–53 of 2020.

**^†^** Hispanic persons could be of any race; American Indian or Alaska Native, Asian, Black, Native Hawaiian or Other Pacific islander, and White persons were non-Hispanic.

## Discussion

During the COVID-19 pandemic in 2020, Black and AI/AN persons had the highest excess all-cause mortality IRs among those aged <25 years and aged 25–64 years, whereas among adults aged ≥65 years, the largest excess mortality IRs occurred among Black and Hispanic persons. These findings underscore the disproportionate prevalence of excess mortality during the COVID-19 pandemic in 2020 among racial/ethnic minority groups of all ages in the United States (*1*–*5*), which have been driven, in part, by factors such as occupational risk, socioeconomic factors, housing conditions, reduced access to health care, and discrimination.^¶¶^ Findings also illustrate the changing impact of the COVID-19 pandemic over time for different subgroups. Among persons aged ≥65 years, excess mortality IRs peaked during April–June 2020 for Black adults, while remaining consistently elevated among Hispanic adults, and increasing from April–June 2020 to October–December 2020 for AI/AN, NH/PI, and White adults. Additional research might help elucidate the factors that contributed to these differences over time.

Recent reports indicate that Black and AI/AN populations experienced the highest age-adjusted death rates in 2020 (*8*), and that the largest percentage excess mortality occurred among racial/ethnic minority groups and persons aged 25–44 years (*6*). This study adds to the literature by describing the excess mortality IRs, which account for population size and age structure. The largest excess mortality IRs occurred among persons aged ≥65 years, with notable differences by race/ethnicity across all age groups. Although excess mortality IRs were lowest among those aged <25 years, there were substantial disparities. Of the nearly 2,000 excess deaths among Black persons aged <25 years, >90% were not directly attributed to COVID-19. Given that injury-related causes of death are typically the leading causes of death among younger age groups, these excess deaths among younger groups and related disparities might be related to increases in homicide, drug overdose, and unintentional injuries in 2020 (*9*).

The findings in this report are subject to at least four limitations. First, estimates of excess mortality might vary when different methods are used for estimating the expected numbers of deaths, and might differ from estimates calculated elsewhere. Second, race/ethnicity reported on the death certificate might be misclassified, resulting in underestimation of rates for some groups (i.e., AI/AN, Asian, and Hispanic populations) (*10*); however, data from NVSS remain one of the most complete sources of race/ethnicity data among public health surveillance systems, with data on race/ethnicity missing for <0.3% of records. Third, data are provisional and subject to change; using more recently published population estimates might also influence the results. Finally, baseline expected counts of deaths were estimated separately for each racial/ethnic group, which might understate total inequities, considering baseline differences in mortality rates by race/ethnicity. If the group with the lowest baseline mortality rates was used as the reference group to estimate excess deaths for all other racial/ethnic groups, then disparities would be even wider.

These findings highlight the importance of timely data to address inequities in social determinants of health that increase the risk for death from COVID-19 among racial/ethnic minority groups. Identifying factors that contribute to racial/ethnic disparities in mortality, either directly or indirectly attributable to COVID-19, can help guide tailored public health prevention strategies and equitable allocation of resources, including COVID-19 vaccination, to achieve greater health equity.

**Summary**

**What is already known about this topic?**

Hispanic or Latino, non-Hispanic Black or African American (Black), and non-Hispanic American Indian or Alaska Native populations have been disproportionately affected by the COVID-19 pandemic.

**What is added by this report?**

Excess mortality incidence rates were higher for persons aged ≥65 years, with notable racial/ethnic disparities across all age groups. In 2020, among Black and Hispanic persons aged ≥65 years, >1,000 excess deaths per 100,000 person-years occurred compared with the number of deaths expected to occur.

**What are the implications for public health practice?**

These findings could help guide targeted public health messaging and mitigation efforts to reduce disparities in COVID-19–associated mortality in the United States, by identifying the racial/ethnic and age groups with the highest excess mortality rates.

## Footnotes

*
https://www.cdc.gov/coronavirus/2019-ncov/prevent-getting-sick/prevention.html

†
https://data.cdc.gov/NCHS/AH-Excess-Deaths-by-Sex-Age-and-Race/m74n-4hbs

§ Deaths from all causes excluding COVID-19 were calculated by subtracting the number of confirmed or presumed COVID-19 deaths from the total number of deaths. Deaths with confirmed or presumed COVID-19 were assigned the ICD-10-CM code U07.1 as a contributing or underlying cause of death on the death certificate. Excess deaths directly attributable to COVID-19 were calculated by subtracting the number of excess deaths from all causes excluding COVID-19 from the total number of excess deaths from all causes.

¶ Observed numbers of deaths were weighted to account for incomplete reporting of provisional death certificate data, where the weights for each jurisdiction were estimated based on the completeness of provisional data from 2019. Weights primarily affected estimates for the most recent 8 weeks, with reported and weighted counts for earlier periods being very similar.

**
https://www.census.gov/data/developers/data-sets/popest-popproj/popest.html

†† sARIMA models were used to estimate weekly population counts and expected numbers of deaths for each race/ethnicity and age group. The cumulative number of excess deaths as of a given week were then subtracted from each subsequent weekly population projection to account for population changes related to the impact of the pandemic. These adjustments were made to improve the accuracy of the population projections by better accounting for smaller increases in population denominators over time because of the greater number of deaths occurring during the COVID-19 pandemic. Without adjustment, the population counts and corresponding expected numbers of deaths might have been overestimated, resulting in underestimates of excess mortality. These adjusted population counts were used to estimate the weekly expected number of deaths for each group by multiplying the expected weekly all-cause mortality IRs (estimated using historical 2015–2019 mortality and population data and sARIMA models accounting for annual temporal and seasonal trends by epidemiologic week) by the weekly adjusted population counts for the period December 29, 2019–January 2, 2021. The resulting weekly expected numbers of deaths were subtracted from the observed numbers of deaths to generate estimates of excess deaths. https://www.medrxiv.org/content/10.1101/2021.02.10.21251461v1.full-text.

§§ 45 C.F.R. part 46, 21 C.F.R. part 56; 42 U.S.C. Sect. 241(d); 5 U.S.C. Sect. 552a; 44 U.S.C. Sect. 3501 et seq.

¶¶
https://www.cdc.gov/coronavirus/2019-ncov/community/health-equity/race-ethnicity.html
